# Advancing musculoskeletal shoulder modeling: reflecting glenohumeral translation with bony, ligamentous, and muscular stability constraints

**DOI:** 10.3389/fbioe.2025.1441530

**Published:** 2025-03-27

**Authors:** Johanna Menze, Eleonora Croci, Michael Skipper Andersen, Hanspeter Hess, Morten Enemark Lund, Enrico De Pieri, Matthias A. Zumstein, Stephen J. Ferguson, Andreas Marc Müller, Annegret Mündermann, Kate Gerber

**Affiliations:** ^1^ School of Biomedical and Precision Engineering, University of Bern, Bern, Switzerland; ^2^ Institute for Biomechanics, ETH Zurich, Zürich, Switzerland; ^3^ Department of Orthopaedics and Traumatology, University Hospital Basel, Basel, Switzerland; ^4^ Department of Biomedical Engineering, University of Basel, Basel, Switzerland; ^5^ Department of Materials and Production, Center for Mathematical Modeling of Knee Osteoarthritis, Aalborg University, Aalborg, Denmark; ^6^ Department of Orthopaedic Surgery and Traumatology Inselspital, Bern University Hospital, University of Bern, Bern, Switzerland; ^7^ AnyBody Technology A/S, Aalborg, Denmark; ^8^ Shoulder, Elbow and Orthopaedic Sports Medicine, Orthopaedics Sonnenhof, Bern, Switzerland; ^9^ Department of Clinical Research, University of Basel, Basel, Switzerland

**Keywords:** shoulder biomechanics, glenohumeral translations, rotator cuff tear, musculoskeletal modelling, force dependent kinematics, ligament modelling

## Abstract

**Introduction:**

Glenohumeral (GH) stability is a delicate interplay between bony congruence, muscle contraction, and ligamentous or capsular stability that can be disrupted by pathologies such as rotator cuff (RC) tears. We aimed to develop an advanced musculoskeletal shoulder model that incorporates subject-specific GH joint contact, active and passive muscle stability, and mechanical properties of ligaments to calculate GH translation using force-dependent kinematics (FDK). We hypothesized that inferior-superior GH translation computed using this model are consistent with *in vivo* GH translation measured by dynamic uniplanar fluoroscopy in healthy shoulders and in shoulders with partial or full RC tears, and that muscle and joint forces computed using the FDK shoulder model are higher than those of the default shoulder model.

**Methods:**

The AnyBody ShoulderArm model was extended to compute GH translation using FDK, considering joint constraints due to bone congruence and to labrum, ligament and muscle stabilization. The inferior-superior GH translations computed using the FDK model were compared with the translations measured using dynamic uniplanar fluoroscopy in healthy shoulders and shoulders with partial and full RC tears during 0°–30° abduction-adduction cycles with 0–3 kg of handheld weight.

**Results:**

The FDK model simulations revealed a decrease in median inferior-superior translations, from 2.8 to 1.8 mm with increasing handheld weight (0–3 kg) which was higher than those observed in fluoroscopic imaging (1.4 mm and 1.1 mm at 0 and 2 kg handheld weight). FDK model simulations in abduction with no additional handheld weight revealed greater variations in glenohumeral translations in shoulders with full RC tear. Compressive joint forces and muscle forces were higher in the FDK model than in the default shoulder model, particularly in the infraspinatus in the healthy model and in the deltoid in the full RC tear model.

**Discussion:**

Distinct differences in muscle and joint forces between the FDK and the default shoulder models confirm that unconstrained translational degrees of freedom of the GH joint are important to advance knowledge of the biomechanical principles of the shoulder. Computed inferior-superior GH translations were greater than *in vivo* measured GH translations, suggesting that joint stability, particularly through muscle recruitment, could be underestimated.

## 1 Introduction

One-third of the general population will experience a rotator cuff (RC) tear in their lifetime (Minagawa et al., 2013). The primary function of the RC is to stabilize the humeral head in the glenoid socket. A lesion can reduce RC muscle strength, resulting in an imbalance of stabilizing forces that can alter glenohumeral (GH) translation and, in severe cases, lead to subacromial impingement (Keener et al., 2009; Melis et al., 2010). An acromiohumeral interval of less than 7 mm, measured on true anterior-posterior radiographs, is indicative of impingement and a severe RC tear with a higher risk of intervention failure (Nové-Josserand et al., 2005; Gruber et al., 2010). The resulting symptoms include reduced range of motion and severe pain (Neer, 1983; Neer et al., 1983). Increased superior GH translations due to RC tears can lead to articular surface degeneration due to increased wear during joint motion, known as RC tear arthropathy, as well as glenoid component loosening in shoulder arthroplasty (Neer et al., 1983; Franklin et al., 1988).

To better understand the reasons for reduced acromiohumeral interval causing poor shoulder function in RC tear and repair, GH translation has been measured using open magnetic resonance imaging (MRI), and uniplanar and biplanar fluoroscopy with and without 3D-to-2D registration of computed tomography (CT)-derived scapula and humerus geometries (Massimini et al., 2012; Graichen et al., 2000; Kijima et al., 2015; Kozono et al., 2018; Giphart et al., 2013; Nishinaka et al., 2016; Chen et al., 1999). Reported inferior-superior GH translation in healthy shoulders varies widely from 1.5 to 5 mm in the inferior or superior direction (Massimini et al., 2012; Graichen et al., 2000; Giphart et al., 2013). Similarly, reported anterior-posterior translation varies from 1 to 6 mm (Massimini et al., 2012).

Changes in GH kinematics due to RC tears have also been studied using uniplanar and biplanar fluoroscopy with and without 3D-to-2D registration of CT with inconclusive results (Kijima et al., 2015; Kozono et al., 2018; Henseler et al., 2014; Yamaguchi et al., 2000). Yamaguchi et al. found significantly greater superior GH translation in patients with symptomatic or asymptomatic RC tears compared with healthy subjects during abduction in the scapular plane from 0° to 150° (Yamaguchi et al., 2000), whereas Kozono et al. and Kijima et al. found no significant differences in inferior-superior GH translation between patients with RC tears and healthy subjects, although they did observe trends towards greater superior GH translation in midrange abduction in patients with RC tears (Kijima et al., 2015; Kozono et al., 2018). Biomechanical studies in cadaveric specimens have confirmed an association of higher GH translations and a resulting higher humeral head subacromial pressure in shoulders with irreparable superior and posterosuperior RC tear compared to intact shoulders (Santos et al., 2023; Muench et al., 2022; Rybalko et al., 2020; Mihata et al., 2016).

GH stability is a delicate interplay between bony congruence, muscle contraction, and ligamentous and capsular stability (Veeger and van der Helm, 2007). A musculoskeletal shoulder model applying these biomechanical properties would enable investigation of the interplay between the various stabilizing components of the shoulder and the disruption of muscular stability due to RC lesions. Stability in translation is primarily confined by the bony congruence and is further increased by the labrum, which increases the concavity depth by 50% (Howell and Galinat, 1989). Resection of the labrum has been shown to reduce the GH stability ratio (ratio of shear to compressive GH joint forces) by 10% (Halder et al., 2001). While muscles provide stability to the humeral head during midrange shoulder motion, ligaments and other connective tissues constrain the joint at the end range of motion (Veeger and van der Helm, 2007). However, most reported musculoskeletal models used to study shoulder function do not account for these stabilizing passive structures and instead define the shoulder as a simple spherical joint (van der Helm, 1994; Garner and Pandy, 2000; Maurel and Thalmann, 1999). The few shoulder models that implement GH translation either define GH joint stiffness based on a reverse engineering approach of measured GH translations (Aurbach et al., 2020) or by defining an overall joint stiffness based on non-linear stiffness function of the inferior GH ligament (Sins et al., 2015).

We aimed to develop an advanced musculoskeletal shoulder model that incorporates an elastic foundation contact model based on subject-specific GH bony morphology, active and passive muscle stability, and passive ligamentous constraints. Using this model, we aimed to (1) compare GH translations using force-dependent kinematics (FDK) (Skipper Andersen et al., 2017) with *in vivo* GH translations measured by dynamic uniplanar fluoroscopy, and (2) compare computed muscle and joint forces between the newly developed FDK shoulder model and the default shoulder model. Firstly, we hypothesised that the inferior-superior GH translation computed by the FDK model would be consistent with *in vivo* GH translation measured by dynamic uniplanar fluoroscopy in healthy shoulders and in shoulders with partial or full RC tears. Secondly, we hypothesised that the computed muscle and joint forces of the FDK shoulder model would be higher than those of the default shoulder model.

## 2 Methods

**FIGURE 1:**
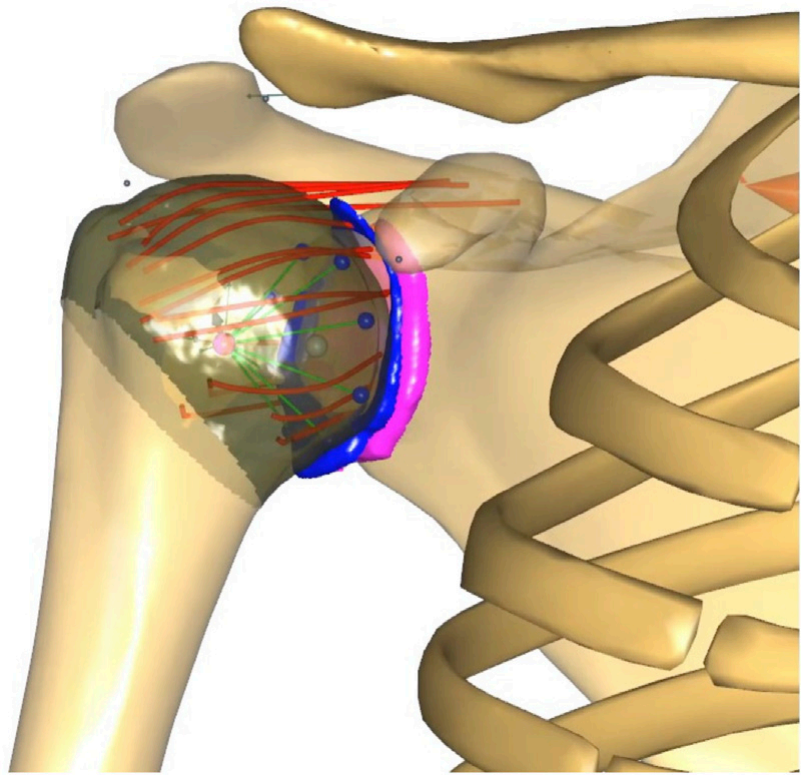
FDK shoulder model with MRI-derived contact surfaces of the glenoid (pink), labrum (blue) and humeral head (brown). The coracohumeral (top) and glenohumeral ligaments, discretised into spring elements (red), originate from the scapula, insert into the humerus and wrap around the humeral head. The original stability constraint (green and blue contact elements conically distributed from the glenoid to the centre of the humeral head) of the default shoulder model was retained.

The generic shoulder model available in the AnyBody™ (AnyBody Modeling System, AMS, version 7.3, Aalborg, Denmark) (Damsgaard et al., 2006) was modified to compute GH translations using FDK, taking into account the joint constraints due to subject-specific bone congruence, as well as the stabilisation provided by the labrum, muscles, and ligaments (Figure 1). The inferior-superior GH translations computed using the FDK model were compared with those measured using dynamic uniplanar fluoroscopy in healthy shoulders and shoulders with partial and full RC tears during 0°–30° abduction-adduction cycles with 0–3 kg of handheld weight. The computed muscle and joint forces of the FDK shoulder model were compared to muscle and joint forces of the default shoulder model.

### 2.1 Participants and data collection

The *in-vivo* data used within this study was collected by Croci et al. with approval of the regional review board (Ethics Committee Northwest Switzerland EKNZ 2021-00182) (Croci et al., 2022). Thirteen right shoulders of participants aged less than 85 years were prospectively enrolled in this study. Inclusion criteria for patients were age between 45 and 85 years with a diagnosed unilateral RC tear involving a partial or full supraspinatus tear with or without injury to other RC muscles. Inclusion criteria for control participants were between 20 and 30 or 45 and 84 years of age, with no history of elbow or shoulder injury and symptoms. Exclusion criteria included a reduced active arm range of motion (less than 30° in abduction and flexion), a documented history of pathology or pain in the contralateral GH joint, a body mass index (BMI) greater than 35 kg/m^2^, history of upper extremity surgery, neuromuscular conditions affecting upper extremity movement, and other pathologies that could affect shoulder joint mobility. Written informed consent was obtained from all participants and the study was conducted in accordance with the principles of the Declaration of Helsinki.

Based on MRI, right shoulders were classified as healthy, partial, or full RC tears, the latter indicating a tear of the entire tendon (Table 2). Body height, body mass, and diagnostic information about the affected shoulder (side, size, severity, and location of the RC tear) were recorded.

#### 2.1.1 Kinematic motion tracking by skin markers

Participants’ shoulder kinematics were recorded using a 3D motion capture system. Retroreflective skin markers were placed on upper body landmarks according to the guidelines of the International Society of Biomechanics (Wu et al., 2005) and recorded by ten infrared cameras sampling at 240 Hz (Vicon Motion System Ltd, Oxford, UK). Clusters of four markers were attached to the acromion and humerus to increase the accuracy of tracking scapulothoracic and GH motion. After static calibration in a seated position, measurements were taken during three bilateral 0°–30° abduction-adduction cycles in the scapular plane, starting from the neutral arm position with the thumb pointing anteriorly. The tasks were performed in an upright seated position with handheld weights of 0–3 kg (1 kg increments) and in a randomised order to eliminate systematic bias due to fatigue. When automatic marker identification failed, manual labelling corrections were performed using the Vicon Nexus software (Version 2.12, Oxford Metrics Inc., Oxford, UK).

#### 2.1.2 Uniplanar fluoroscopy measurement of glenohumeral translations

Dynamic uniplanar fluoroscopic imaging (Multitom Rax, Siemens Healthineers, US) was used to capture single abduction-adduction cycles in the scapular plane ranging from 0° to 30° with and without a 2 kg handheld weight at a 10 Hz pulse rate. These cycles were performed under similar conditions to those recorded with the skin-mounted marker measurement. Image dimensions were calibrated using a 25 mm reference sphere. To measure GH translations, we registered the humeral head centre, the humeral shaft, the most lateral point of the acromion, and the inferior and superior glenoid edges. The humeral head centre was defined as the geometric centre of the articular surface of the humeral head (Schröter et al., 2016; Verstraeten et al., 2013; Jacxsens et al., 2015). Subsequently, GH translations during arm abduction and adduction were measured in the glenoid coordinate system (Teyhen et al., 2010; Croci et al., 2023a).

#### 2.1.3 Glenohumeral geometries from MRI

MRI scans were acquired using a 3T scanner (Prisma, Siemens Healthineers, US) with dedicated shoulder and body array coils. No contrast agent was administered to the participants. From the applied MRI protocol (Croci et al., 2023b), a fat-saturated transverse proton density turbo spin echo (TSE) sequence and a coronal T2-weighted BLADE sequence were used to extract subject-specific geometries of the glenoid, humeral head and labrum. The structures were automatically segmented in both planes using a trained artificial intelligence model (nn-Unet) (Isensee et al., 2021) and then interpolated to obtain high-resolution geometries. A clinical expert reviewed the segmentation quality and manually corrected it as needed (ITK-Snap 3.6.0). A Laplace smoothing filter was applied to the generated surface models (MeshLab 2022.02) (Sorkine, 2005). The geometry of the humeral head, labrum and glenoid were isolated and reduced to the surface facing the GH joint by selecting the corresponding vertices (MeshLab 2022.02).

### 2.2 Musculoskeletal modelling

The motion capture data were used as input for an inverse dynamics analysis in the AMS. Models of the right shoulder were scaled to match each participant’s anthropometrics (height and weight) and the marker data during a seated reference trial (Croci et al., 2022). A kinematic analysis based on the marker trajectories was conducted to compute primary joint kinematics (Andersen et al., 2009; Lund et al., 2015). An inverse dynamics analysis based on a third-order polynomial muscle recruitment criterion was then performed to calculate the active muscle forces required to perform the given input motion and the resulting joint contact forces.

#### 2.2.1 FDK shoulder model

The previously constrained translational degrees of freedom (DOF) of the GH joint in the ShoulderArm model, available in the AnyBody Managed Modelling Repository (AMMR) v. 2.2.3, were modified to be force-dependent. In addition to the primary joint kinematics, which are driven by external loads and muscle forces, the GH translations, or secondary kinematics, were then driven by a quasi-static equilibrium of forces, including muscle forces, passive ligament forces and contact forces between the humeral head, labrum, and glenoid surfaces. For each time frame of the modelled shoulder motion, the FDK solver computes the position of the humeral head with respect to the glenoid that best achieves an equilibrium between muscle, ligament, bone contact, and external forces. An acceptable minimum FDK residual force threshold of 10 N was defined (Skipper Andersen et al., 2017).

#### 2.2.2 Bone contact surfaces

The humeral head to glenoid and humeral head to labrum contact models were implemented using an elastic foundation contact model based on subject-specific surfaces. The generic (scaled) humeral head and glenoid bony surfaces were exported from the AMS and rigidly registered to the subject-specific glenoid and humeral head orientation using inertial alignment registration (Mimics Medical 20.0, Materialise, Leuven, Belgium). Reverse registration was then applied to align the subject-specific partial surfaces onto the generic (scaled) bone morphologies in the AMS musculoskeletal models. Contact forces were computed using an elastic foundation contact model, by multiplying the penetration volume by a material-dependent pressure module. We defined the pressure module of the glenoid and humeral head bone as 9.3 × 10^9^ N/m^3^ and that of the humeral head to the labrum as 0.11 × 10^9^ N/m^3^ (Marra et al., 2015; Carey et al., 2000). The latter was based on results from the labral compressive modulus, the Poisson ratio of the meniscus and the equation for calculating the deformation response of a thin bonded compressible elastic layer (Carey et al., 2000; Danso et al., 2018; Argatov and Mishuris, 2015).

#### 2.2.3 Glenohumeral and coracohumeral ligaments

The superior, middle and inferior GH and coracohumeral ligaments were included in the FDK shoulder model to simulate the stability that these structures provide to the GH joint, specifically at the end of range of motion (Figure 1). Ligament bundles were defined by connecting origin and insertion sites via a spherical humeral head wrapping surface. The origin and insertion sites were based on anatomical landmarks of the generic bony surfaces in the AMS taken from (Motsinger, 2020) The superior, middle and inferior GH and the coracohumeral ligaments were discretised into two, two, eight and five individual spring elements, respectively, to account for the distribution of insertion sites and wrapping width. The mechanical properties of the ligaments were simulated as nonlinear elastic elements with a slack length, a toe region and a linear elastic region as proposed by Marra et al. (2015). We defined the elastic stiffness of each ligament bundle based on published experimental data (Table 1; Bigliani et al., 1992; Boardman et al., 1996). A ligament length calibration was performed for each subject before the inverse dynamic analysis by defining the individual slack lengths in GH positions with known ligament strains (Table 1; Amadi et al., 2012; Urayama et al., 2001; Brenneke et al., 2000).

**TABLE 1:** Mechanical properties of the glenohumeral (GH) and coracohumeral ligaments and modelled shoulder position used to calibrate ligament strain and slack length.

Ligament	Elastic stiffness (N/-)	Shoulder position	Ligament strain
Inferior GH (posterior)	320 (Bigliani et al., 1992; Boardman et al., 1996; Amadi et al., 2014; Southgate, 2009)	60° abduction in scapular plane 30° external rotation	10% (Amadi et al., 2012; Urayama et al., 2001; Brenneke et al., 2000)
Inferior GH (inferior)	388 (Bigliani et al., 1992; Boardman et al., 1996; Amadi et al., 2014; Southgate, 2009)	60° abduction in scapular plane 80° internal rotation	10% (Amadi et al., 2012; Urayama et al., 2001; Brenneke et al., 2000)
Inferior GH (anterior)	582 (Bigliani et al., 1992; Boardman et al., 1996; Amadi et al., 2014; Southgate, 2009)	60° abduction, 50° external rotation	11% (Amadi et al., 2012; Urayama et al., 2001; Brenneke et al., 2000)
Middle GH	375 (Bigliani et al., 1992; Boardman et al., 1996; Amadi et al., 2014; Southgate, 2009)	0° abduction, 0° axial rotation	7% (Amadi et al., 2012; Urayama et al., 2001; Brenneke et al., 2000)
Superior GH	550 (Bigliani et al., 1992; Boardman et al., 1996; Amadi et al., 2014; Southgate, 2009)	0° abduction, 20° external rotation	9% (Amadi et al., 2012; Urayama et al., 2001; Brenneke et al., 2000)
Coracohumeral	1099 (Bigliani et al., 1992; Boardman et al., 1996; Amadi et al., 2014; Southgate, 2009)	0° abduction, 0° axial rotation	5% (Amadi et al., 2012; Urayama et al., 2001; Brenneke et al., 2000)

#### 2.2.4 Shoulder muscles

Sixteen muscles spanning the shoulder joint were discretised into 118 muscle elements to achieve more anatomical muscle lines of action. Muscle modelling for GH motion and stabilisation was performed using the characteristics of the three-element Hill muscle model (Hill, 1953). In this model, strength depends not only on the physiological muscle cross-sectional area, but also on the instantaneous muscle fibre length and contraction velocity. For each subject, the operating range of each shoulder muscle was calibrated throughout the full range of motion of the shoulder (0°–180° shoulder abduction, 0°–160° shoulder flexion, −70°–90° internal to external rotation) to match that defined by Garner and Pandy (Garner and Pandy, 2003). The strength of the torn RC muscles (supraspinatus, infraspinatus and or subscapularis) was inactivated to simulate a full-thickness RC tear and reduced to 50% strength to simulate a partial RC tear.

#### 2.2.5 Glenohumeral stability constraint

To achieve muscular stabilisation of the GH joint, especially in the midrange of motion, the shoulder stability criterion, a default constraint in the Anybody shoulder model, was retained. This criterion requires the joint reaction force vector to fall within the glenoid cavity. To avoid interference between the contact simulation of the subject’s GH joint surfaces and the modelled stability constraint, an additional weightless GH segment was constructed to connect the two GH joint constraints.

### 2.3 Data analysis

For each patient, the mean course of GH translations, and mean muscle, joint and ligament forces were derived from the three repetitions of 0°–30° abduction-adduction cycles.

The GH translations computed with the FDK model were compared between abduction-adduction cycles with different handheld weights and with the measured inferior-superior translations from dynamic fluoroscopy imaging of the same subject. Predicted anterior-posterior GH translations and maximum ligament forces and strains computed with the FDK model were compared between healthy, partial and full RC tear shoulders.

**FIGURE 2:**
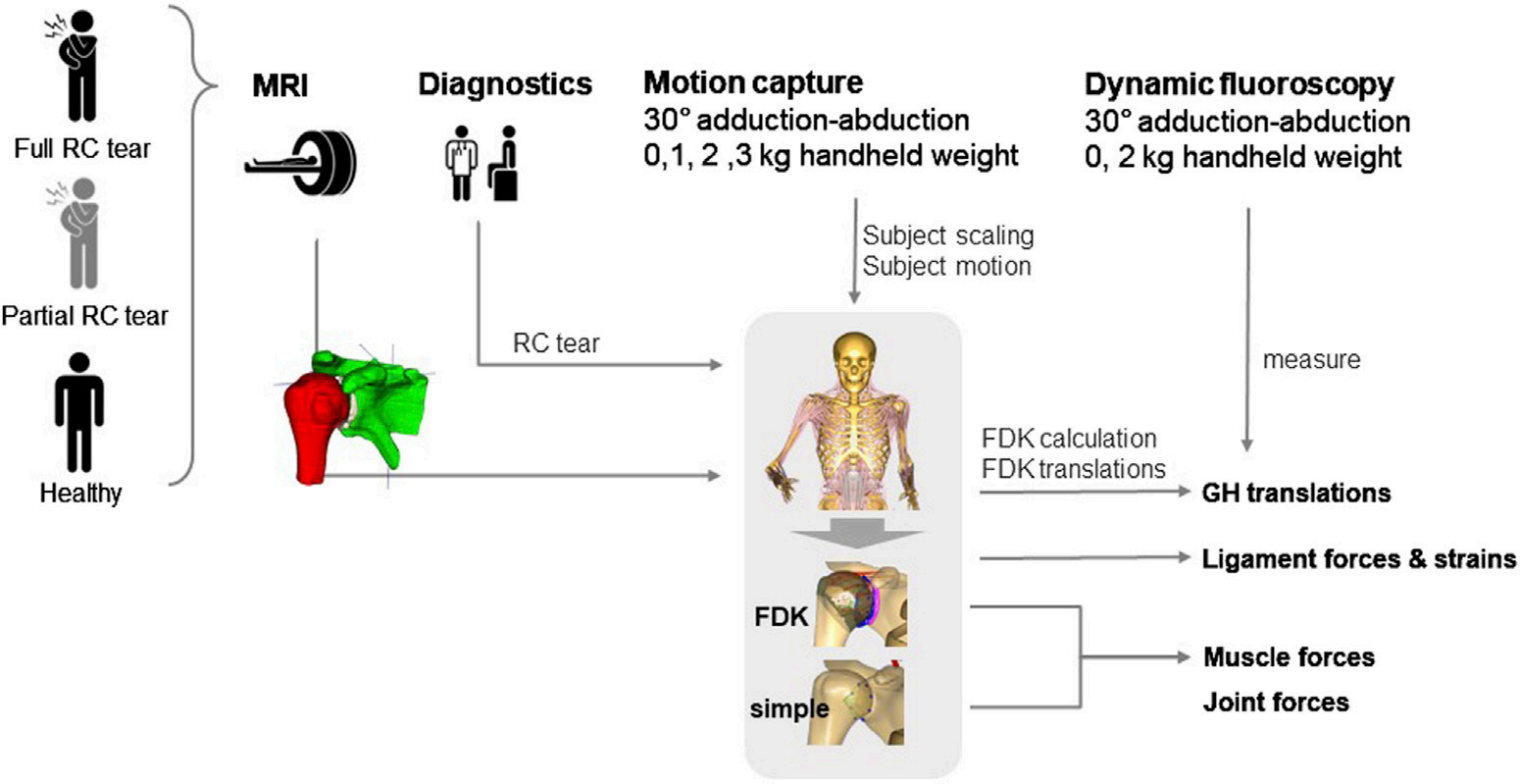
Schematic overview of data acquisition in healthy, partial and full rotator cuff (RC) tear shoulders for model personalisation and outcome analysis. MRI, magnetic resonance imaging; FDK, force-dependent kinematics; GH, glenohumeral.

Furthermore, for each participant, the predictions of the default generic shoulder model from the AMS and the custom FDK shoulder model with subject-specific bone contact and ligament structures were compared. Maximum muscle forces and joint forces at maximum GH abduction angle were compared between healthy, partial and full RC tear shoulders and between the FDK and default shoulder (Figure 2).

## 3 Results

Results were compared between four healthy shoulders, seven partial and two full RC tear shoulders (Table 2). While simulations of three abduction-adduction cycles were completed for all shoulders with 0 kg of handheld weight, five, five and seven simulations failed to converge with 1, 2, and 3 kg handheld weight during abduction-adduction cycles, respectively (Table 2).

**TABLE 2:** Shoulders grouped into healthy, partial and full rotator cuff (RC) tear, details of partial (p.) or full thickness supraspinatus (supras.), infraspinatus (infras.) or subscapularis (subscap.) tears and information on completion of simulation (tick) per simulation with 0–3 kg handheld weight.

Shoulder	Group	Affected RC	0 kg	1 kg	2 kg	3 kg
1	Healthy	—	✓	✓	✓	✓
2	Healthy	—	✓	✓		
3	Healthy	—	✓	✓	✓	✓
4	Healthy	—	✓	✓	✓	
5	Partial RC tear	Supras.	✓			
6	Partial RC tear	Supras.	✓			
7	Partial RC tear	Supras.	✓		✓	✓
8	Partial RC tear	Supras.	✓	✓		✓
9	Partial RC tear	Subscap.	✓	✓	✓	✓
10	Partial RC tear	Supras. Infras. Subscap.	✓	✓	✓	
11	Partial RC tear	Supras. Infras. Subscap.	✓	✓		
12	Full RC tear	Subscap. Supras. (p)	✓		✓	✓
13	Full RC tear	Supras. Infras. Subscap. (p)	✓			

### 3.1 Glenohumeral translations

**FIGURE 3:**
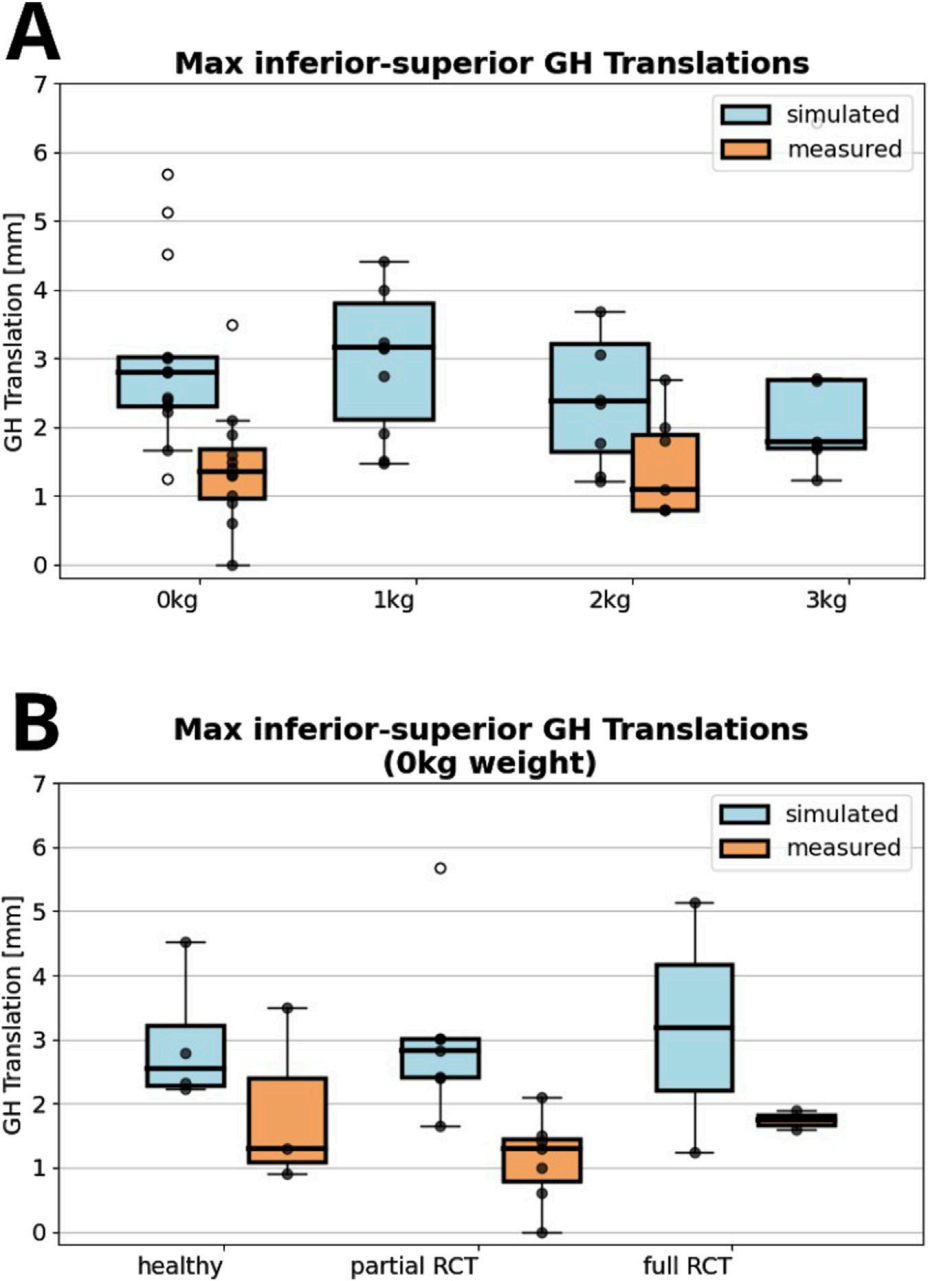
**(A)** Maximum absolute value of computed inferior superior glenohumeral (GH) translations with zero to 3 kg of handheld weight of all shoulders compared to fluoroscopy measured glenohumeral translations. **(B)** Comparison between fluoroscopically measured and simulated glenohumeral translations for healthy shoulders, partial or full rotator cuff (RC) tear shoulders for 0°–30° adduction-abduction cycles. All data points are shown with outliers indicated as non-filled markers.

**FIGURE 4:**
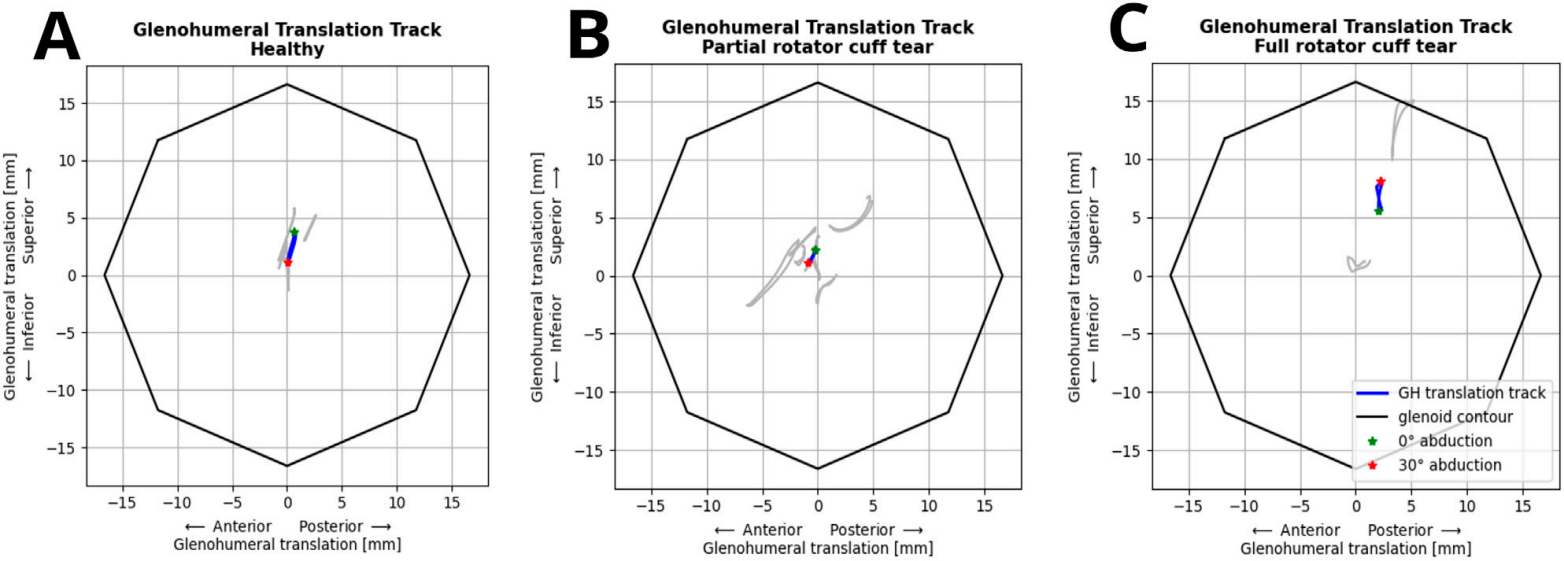
Individual (grey) and mean glenohumeral head translation path (blue) of shoulders during abduction-adduction cycles from 0° to 30° without handheld weight relative to the glenoid contour in the healthy **(A)**, partial **(B)** and full **(C)** rotator cuff tear shoulders.

The FDK model simulations revealed a decrease in median inferior-superior translations, from 2.8 to 1.8 mm with increasing handheld weight (0–3 kg) which was higher than those observed in fluoroscopic imaging (1.4 mm and 1.1 mm at 0 and 2 kg handheld weight) (Figure 3A). FDK model simulations in abduction with no additional handheld weight revealed greater variations in GH translations in shoulders with full RC tear compared to healthy shoulders (Figure 3B). The GH translation path was directed inferior and anterior in the healthy and partial RC tear shoulders (Figure 4). The differences between GH translation paths within a group increased with increasing RC tear severity. The mean translation path was smaller in the partial RC tear group because translations were directed inferiorly and superiorly. In the full RC tear shoulders, the mean translation path was directed superiorly. In the right shoulder of subject 13 (full supraspinatus, infraspinatus, partial subscapularis tear), the computed humeral head position was more superior than in the other shoulders and translations were greatest.

### 3.2 Muscle forces

**FIGURE 5:**
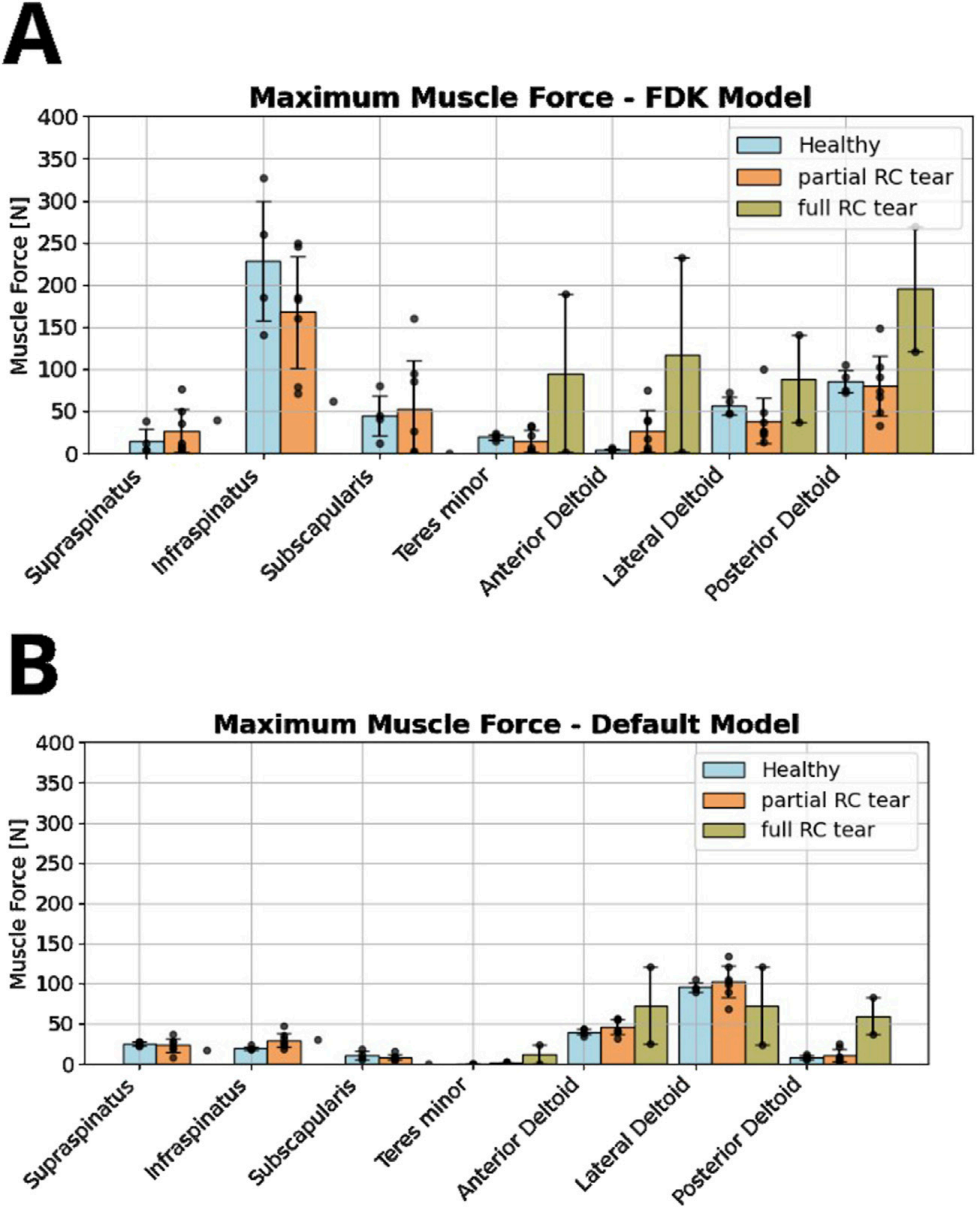
Comparison of rotator cuff and deltoid muscle forces between healthy, partial and full RC tear shoulders during abduction motion without a handheld weight, computed in the force-dependent kinematic (FDK) model **(A)** and the default shoulder model **(B)**.

In the FDK model simulations of healthy participants, the highest muscle forces were computed for the infraspinatus (228 ± 71 N), followed by the lateral and posterior deltoid, and the subscapularis and teres minor (Figure 5A). Using the FDK model, shoulders with a full RC tear had minimal rotator cuff forces and greater anterior, lateral and posterior deltoid forces compared to the healthy model (117 ± 116 N, 89 ± 52 N and 195 ± 74 N *versus* 4 ± 1 N, 57 ± 11 N and 86 ± 13 N, respectively). Compared to the default shoulder model, the FDK model computed higher infraspinatus forces in the healthy group and higher posterior deltoid and teres minor forces in the full RC tear group (Figures 5A, B). The computed anterior and lateral deltoid forces of the healthy group were lower with the FDK shoulder model compared to the default model (Figure 5B).

### 3.3 Ligament forces and strains

In all shoulders, the highest ligament forces were computed for the middle GH ligament (45–74 N), followed by the anterior portion of the inferior GH ligament (46–63 N) and superior GH ligament (19–55 N) (Table 3). Forces and strains were lower in the partial and full RC shoulders compared to the healthy shoulders. Shorter ligament lengths, i.e., unstrained ligaments (negative strain) were simulated for the inferior and posterior regions of the inferior GH ligament such that they did not carry any load in the healthy and RC tear shoulders. The coracohumeral ligament was strained from 3% to 13% in the different shoulders resulting in forces between 11 and 25 N.

**TABLE 3:** Mean ± standard deviation maximum forces (N) and strains (%) in the superior, middle, inferior glenohumeral (GH) and coracohumeral ligaments of the healthy, partial and full rotator cuff (RC) tear shoulders during 0° to 30° abduction-adduction without handheld weight. The inferior GH ligament is divided into an anterior, posterior and inferior portions.

Ligament		Healthy (N = 4)	Partial RC tear (N = 6)	Full RC tear (N = 3)
Superior GH	Force (N)	55 ± 17	45 ± 24	19 ± 15
	Strain (%)	23 ± 6	19 ± 9	8 ± 6
Middle GH	Force (N)	74 ± 12	66 ± 21	45 ± 3
	Strain (%)	43 ± 6	38 ± 11	27 ± 1
Inferior GH (anterior)	Force (N)	63 ± 15	56 ± 30	46 ± 9
	Strain (%)	24 ± 6	21 ± 12	15 ± 7
Inferior GH (inferior)	Force (N)	0	0	0
	Strain (%)	−39 ± 2	−32 ± 11	−41 ± 13
Inferior GH (posterior)	Force (N)	0	0	0
	Strain (%)	−49 ± 3	−46 ± 10	−52 ± 6
Coracohumeral	Force (N)	25 ± 10	19 ± 10	11 ± 11
	Strain (%)	13 ± 6	9 ± 6	3 ± 9

### 3.4 Joint reaction forces

Measured compressive and shear joint reaction forces at maximum GH abduction angle varied more using the FDK model than the default shoulder model (Table 4), with clear differences in the overall trends. Compressive joint reaction forces were greater with the FDK model than with the default shoulder model for all shoulders. In both models, there was an increase in compressive forces in the full RC tear shoulders compared to the healthy shoulders (FDK: 236–331 N, default: 104–135 N). The inferior-superior shear forces in the FDK model were lower than in the default model. In the FDK model, the variance in superior-inferior shear forces was high in the full RC tear group, ranging from inferior to superior shear forces, especially in the full RC tear group (−18 ± 73 N). While the anterior-posterior shear forces were in a similar range between the FDK and the default shoulder models for the healthy and partial RC tear shoulders, the anterior shear forces were higher in the full RC tear group (225 ± 176 N) compared to the healthy (26 ± 9 N) and partial RC tear shoulders (25 ± 35 N) and compared to the anterior-posterior shear forces in the default model (47 ± 15 N).

**TABLE 4:** Mean ± standard deviation glenohumeral (GH) joint forces (N) calculated at maximum abduction angle for each group (healthy, partial and full rotator cuff (RC) tears). Compressive force and anterior-posterior (AP) and inferior-superior (IS) shear forces are shown.

Force (N)	Model	Healthy (N = 4)	Partial RC tear (N = 6)	Full RC tear (N = 3)
Compressive (comp. +)	FDK	236 ± 94	182 ± 47	331 ± 245
	Simple	104 ± 5	135 ± 34	132 ± 12
IS shear (superior +)	FDK	1 ± 21	39 ± 63	−18 ± 73
	Simple	97 ± 10	103 ± 18	122 ± 29
AP shear (anterior +)	FDK	26 ± 9	25 ± 35	225 ± 176

## 4 Discussion

We herein present an advanced musculoskeletal shoulder model that incorporates the subjects’ anatomical GH joint bony contact, active and passive muscle stability and ligament forces to compute GH translation using FDK.

Comparison of simulated inferior-superior GH translations showed greater variations of translations in shoulders with full RC tears compared to shoulders with partial RC tears and healthy shoulders, with the humeral head translating more superiorly in the RC tear shoulders and inferiorly in the healthy shoulders. A general trend of anterior GH translations was observed in all shoulders, which is consistent with the GH translations measured from biplanar imaging of Giphart et al. (2013) and Zhang et al. (2016). The more superior position and superior migration of the humeral head close to the glenoid rim in the subject with a massive RC tear involving the supraspinatus, infraspinatus and partial subscapularis is consistent with the decreased acromiohumeral distance observed clinically in shoulders with severe RC tears (Nové-Josserand et al., 2005; Walch et al., 1992). The association of tear shape and location and direction of GH translation as found by Santos et al. will be investigated with future data of additional shoulders (Santos et al., 2023). Computed inferior-superior GH translations in the FDK shoulders were generally greater than the measured translations in dynamic fluoroscopy. Computed translations decreased with increasing handheld weight during the abduction-adduction cycles but remained greater than measured translations.

Greater RC muscle forces were required for abduction with greater handheld weights, indicating that forced muscle recruitment resulted in a stabilizing effect on the GH joint. The larger simulated GH translations compared to the measured data and the high number of model failures, mainly due to superior escape of the humeral head, indicate that the GH stability was underestimated in the presented model. Williamson et al. similarly concluded for cadaveric shoulder studies that RC muscle activation is necessary to realistically simulate GH kinematics (Williamson et al., 2020). The importance of muscle stability has also been highlighted by Kronberg et al. who compared the shoulder muscle activity between patients with general joint laxity and healthy controls (Kronberg et al., 1991).

Muscle forces computed with the FDK model clearly differed from those computed with the default shoulder model. On average, greater forces were observed in the posterior shoulder muscles using the FDK model compared to the default shoulder model, particularly in the infraspinatus and teres minor in the healthy and partial RC tear shoulders and in the posterior deltoid in the full RC tear shoulders. The posterior deltoid appeared to compensate for the compromised supraspinatus and infraspinatus in the full RC tear shoulders. Greater deltoid forces with infraspinatus and supraspinatus tears were also reported by Steenbrink et al., but they did not differentiate between the different parts of the deltoid (Steenbrink et al., 2009). The higher infraspinatus and higher subscapularis forces in the FDK model compared to the default shoulder model may reflect the need for additional stabilization of the GH joint with respect to the unconstrained translational DOFs. Clinically, the infraspinatus and subscapularis are known as the GH force couple that stabilizes the humeral head in the glenoid cavity (Payne et al., 1997; Piepers et al., 2014). The greater forces observed in the infraspinatus compared to the subscapularis, could be a consequence of its more agonistic function for abduction, but could also be a counterbalance to the additional stability provided by the anterior ligaments.

While the inferior and posterior portions of the inferior GH ligament remained slack during the simulation, and therefore did not bear any load, the coracohumeral, middle and superior GH ligaments and the anterior portion of the GH ligament were elongated, reaching forces of up to 74 N and 42% elongation. Massimini et al. used an *in vivo* MR imaging technique to measure GH ligament elongation and reported 25%, 70% and 105% ligament elongation for the superior and middle GH ligaments and the anterior bands of the inferior GH ligament and a shortening of the posterior band of the inferior GH ligament at 45° shoulder abduction (Massimini et al., 2012). While our results were consistent with the overall trend reported by Massimini et al., the ligaments were more elongated in the FDK shoulder model after inverse dynamics simulation than initially calibrated in the static arm positions based on the literature (Table 1; Massimini et al., 2012; Urayama et al., 2001; Brenneke et al., 2000). Adjusted humeral head positions and translations computed to achieve force equilibrium using FDK could be the cause of this discrepancy, as ligament elongation is highly sensitive to changes in humeral head position, especially for short ligament lengths. The lower ligament elongation in RC tear shoulders compared to the healthy shoulders may be a result of the more anterosuperior humeral head position. Amadi et al. reported ligament forces of a similar magnitude, with middle GH ligament forces of 40–60 N with 4–6 mm GH translations simulated in anterior and inferior laxity tests and no loading in the posterior inferior GH ligaments (Amadi et al., 2012). However, the large increase in infraspinatus forces observed in our study may indicate that the ligament forces were too high. As the GH ligaments are mainly composed of collagen fibres, a maximum strain of 3%–7% can be reached without lesion, which is well below our reported strains (Malicky et al., 2002). To our knowledge, this is the first study to implement physiological ligaments in a musculoskeletal model. In this study, ligament insertion points, stiffness and slack length were based on generic definitions. The sensitivity of the model results to the stability balance between muscles and ligaments and the robustness of the ligament properties should be evaluated in a future sensitivity study.

Compressive joint forces were significantly higher in the FDK model compared to the default shoulder model. The additional RC muscle forces and ligament forces in the FDK model stabilized the humeral head in the glenoid, thereby increasing the compressive joint forces. As the humeral head migrated both inferiorly and superiorly, the overall inferior-superior shear forces were small with a large variability. The anterior-superior shear force remained low in the healthy and partial RC tear shoulders but increased significantly in the full RC tear shoulders compared to the healthy and partial RC tear shoulders. The anterior-posterior force couple was influenced by the RC lesion, especially with full infraspinatus tear, which led to higher anterior shear. The computed GH joint forces were in the range of *in vivo* joint reaction forces measured with instrumented prostheses in five shoulders (200–400 N total force at 30° abduction) (Bergmann et al., 2011) and in the range of simulated GH joint forces between different shoulder models (20%–40% body weight) (Prinold et al., 2013). In general, high intra-group variability in joint forces were observed, especially in the FDK model, reflecting a large heterogeneity between RC tear patterns in the shoulders and the overall small sample size. Data from additional shoulders will be modelled to better understand the influence of specific RC tear patterns on joint forces.

To the best of our knowledge, this is the first musculoskeletal model to include a physiological implementation of the coracohumeral and GH ligaments and additional consideration of subject-specific joint surfaces and motion. We hypothesize that a few deficiencies in the used models may have contributed to the overestimation of translations. First, cartilage was not considered, but cartilage increases GH joint congruence, potentially contributing to GH stability and reducing GH translations (Lippitt and Matsen, 1993; Flatow et al., 1991). Second, the patient specific geometry of the scapula was not included in the model due to the limited field of view of the clinical routine MRI used. Therefore, muscle insertion points, as well as subject-specific acromion and glenoid orientation, could not be considered. However, glenoid orientation and acromion lateralization contribute to stability of the GH joint, as shear and compressive forces are directly dependent on these morphological measures (Moor et al., 2016; Villatte et al., 2020). Therefore, in future advancements of the model, the cartilage surface will be additionally modeled and the full subject-specific scapula may be imaged to apply the automatic scapula morphing method of Oswald et al. in the FDK shoulder model (Oswald et al., 2023). Ligament insertion points, stiffness and slack length were defined generically based on anatomical and experimental studies as they cannot be derived from imaging. However, subject-specific ligament laxity affects the stability of the GH joint and may lead to GH dislocation. The inferior-superior GH translations used to verify the FDK model were measured from uniplanar fluoroscopic images and thus may have been affected by projection error. We recommend that dynamic biplanar radiographic imaging could be used for more accurate verification in future studies, and that anterior-posterior GH translations should also be compared (Millett et al., 2016). Clinically, computed GH translations and humeral head positions given the acting joint, muscle and ligament forces provide a biomechanical explanation for various shoulder pathologies. In the future, RC tear treatment using subject-specific musculoskeletal shoulder models can become more effective by planning targeted muscle strengthening to reduce GH translations. Subject which are prone to traumatic shoulder instability could be identified before a humeral head dislocation and preventive shoulder strengthening training and physiotherapy could be performed. A future shoulder model that reliably reflects shoulder instability and stabilizes structures will further help preoperative planning of shoulder arthroplasty, RC repair and tendon transfer.

## 5 Conclusion

Distinct differences in muscle and joint forces between the FDK and the default shoulder model confirm that unconstrained translational DOF of the glenohumeral joint is important for advancing knowledge of the biomechanical principles of the shoulder and to allow for subject-specific treatment planning based on musculoskeletal modeling in the future. Inferior-superior GH translations computed with this model were greater than *in vivo* GH translations measured by dynamic uniplanar fluoroscopy in healthy shoulders and in shoulders with partial or full RC tears indicating joint stability, particularly through muscle recruitment, is currently underestimated.

## Data Availability

The raw data supporting the conclusion of this article will be made available by the authors, upon reasonable request.
